# ﻿A new species and three newly recorded species of Tetrastichinae (Hymenoptera, Eulophidae) from China

**DOI:** 10.3897/zookeys.1131.90688

**Published:** 2022-11-24

**Authors:** Wen-Jian Li, Cheng-De Li

**Affiliations:** 1 Jiangsu Provincial Key Laboratory of Coastal Wetland Bioresources and Environmental Protection, School of Wetland, Yancheng Teachers University, Yancheng, 224007, China; 2 Jiangsu Key Laboratory for Bioresources of Saline Soils, School of Wetland, Yancheng Teachers University, Yancheng, 224007, China; 3 School of Forestry, Northeast Forestry University, Harbin, 150040, China

**Keywords:** Chalcidoidea, genera, parasitoids, taxonomy

## Abstract

Five species of five genera in Tetrastichinae (Hymenoptera, Eulophidae) from China are reviewed, including one new species, *Mestocharellaqingdaoensis***sp. nov.**, and three new country record species: *Nesolynxthymus* (Girault, 1916), *Holcotetrastichusrhosaces* (Walker, 1839), and *Peckelachertusdiprioni* Yoshimoto, 1970. New distributional data for *Ceratoneuraindi* Girault, 1917 are provided.

## ﻿Introduction

The subfamily Tetrastichinae (Hymenoptera, Eulophidae) is one of the largest groups of Chalcidoidea (Graham 1987; LaSalle 1994). Species are distributed in almost all geographic realms and play a vital role in terrestrial ecosystems (Graham 1987; LaSalle 1994). Most species of Tetrastichinae are parasitic; they attack species from approximately 1000 families in 10 different orders of Insecta (Graham 1987; LaSalle 1994). Also, some species, such as *Leptocybeinvasa* Fisher and LaSalle, 2004, are phytophagous and live in galls produced by their hosts.

Unfortunately, Chinese species of Tetrastichinae are poorly investigated compared to other countries and regions (Kostjukov 1978, 2000; Graham 1987, 1991; Bouček 1988; LaSalle 1994; Narendran 2007). In the early stage, foreign entomologists reported several Tetrastichinae from Guangdong, Macao, and Taiwan in China (Perkins 1912; Timberlake 1921; Miwa and Sonan 1935). With more research on parasitic wasps, Chinese entomologists realized the importance of this faunal group: Liao et al. (1984) reported 201 economically important insect species of China including eight species of Tetrastichinae; Yang (1996) systematically investigated parasitic wasps on bark beetles from China and reported 141 species including 16 species of Tetrastichinae; Zhu and Huang (2001) investigated Eulophidae from Zhejiang province and reported 12 species of Tetrastichinae; Zhu and Huang (2002) investigated Eulophidae from Guangxi province and reported 23 species of Tetrastichinae; Zhang et al. (2007) investigated Eulophidae from south Gansu and Qinlin Mountains and reported 14 species of Tetrastichinae; Yang et al. (2015) systematically investigated parasitic wasps on forest defoliators, reporting 115 species including 20 species belonging to four genera of Tetrastichinae. Subsequently, there are many more reports of new species and records of Tetrastichinae (Yang 1989; Sheng and Wang 1992, 1993; Sheng 1995; Sheng and Zhao 1995; Sheng and Zhu 1998; Yang and Wei 2003; Jiao et al. 2006; Zhang et al. 2009; Wu et al. 2009; Li et al. 2014; Yang et al. 2014; Feng et al. 2016; Li et al. 2016; Song et al. 2017; Li and Li 2020, 2021; Song et al. 2020; Guo et al. 2022; Ning et al. 2022). In terms of Tetrastichinae species richness, there is an obvious imbalance among provinces of China. Most southern provinces have more species than northern provinces, such as 27 species in Guangxi Province compared with just two species in Ningxia Province. Therefore, there is still much to study, and knowledge to be gained, about this group in China.

## ﻿Materials and methods

Specimens were collected by sweep netting and yellow-pan trapping. They were preserved and were dissected and mounted in Canada balsam following the method of Noyes (1982), or fixed on triangular cards. Photographs were taken with a digital CCD camera attached to an Olympus BX51 compound microscope and a AOSVI HK-830 microscope. Most measurements were made from slide-mounted specimens using an eye-piece reticule with an Olympus CX21 microscope. Terminology follows Gibson et al. (1997) and the following abbreviations are used:

**F1–4** (flagellomeres 1–4);

**POL** (minimum distance between lateral ocelli);

**OOL** (minimum distance between lateral ocellus and eye margin);

**OD** (longest diameter of a lateral ocellus);

**MV** (marginal vein);

**STV** (stigmal vein);

**SMV** (submarginal vein);

**PMV** (postmarginal vein).

All the specimens listed below are deposited in Northeast Forestry University (**NEFU**), Harbin, China.

## ﻿Species accounts

The genus *Mestocharella* (Eulophidae, Tetrastichinae) was erected by Girault (1913) with *Mestocharellaferalis* Girault, 1913 as the type species. It is a small genus with 12 valid species worldwide (Noyes 2019) and only one species occurring in China, *M.javensis* (Kamijo 1994). Because the propodeum is different from the propodeum of all the other species included in *Mestocharella*, *M.deltoids* Khan, Agnihotri & Sushil, 2005 and *M.indica* Jaikishan Singh & Khan, 1995 probably do not belong to the genus (Narendran 2007).

*Mestocharella* is a unique genus and can be distinguished from Tetrastichinae by the following characteristics: malar sulcus present; antenna slender, one anellus, funicle with four segments and clava bi-segmented in female; funicle 4-segmented and clava 3-segmented in male; pronotum long, collar with or without transverse carina; axillae not so advanced; dorsellum with a median carina; propodeum long, with a large subpentagonal area; spiracles small; gastral petiole conspicuous, strongly carinate; gaster usually shorter than mesosoma.

The species of *Mestocharella* can be divided into three species groups: the *kumatai*, *feralis*, and *javensis* groups (Kamijo 1994). The species of *Mestocharella* are parasitic on Lepidoptera (Bouček 1988; Kamijo 1994).

### ﻿ Mestocharella qingdaoensis sp. nov.

Taxon classificationAnimaliaHymenopteraEulophidae

CC48855D-5A51-5BB2-A205-5243138C7F25

https://zoobank.org/BA195019-145C-4A7E-838E-BAEB094AF9FB

Figs 1–4

#### Type material.

***Holotype***, female [on card], China, Shandong Province, Qingdao City, Mount Xiao Zhu, 18–20.V.2014, Guo-Hao Zu, Si-Zhu Liu, by yellow pan trapping (deposited in NEFU). ***Paratypes***,1 female [on slide], same data as holotype (deposited in NEFU).

#### Diagnosis.

Female. Body mainly brownish, head and posterior half of mesoscutum and axillae yellow; propodeum median carina not forked anteriorly; plicae distinct but not connected with median carina; forewing SMV with three dorsal setae, MV 6.9–7.3× as long as STV. *Mestocharellaqingdaoensis* belongs to the *kumatai* group (Kamijo 1994) in that the pronotal collar is without transverse carina, and it is similar to *M.kumatai* Kamijo, 1994. However, it can be separated from *M.kumatai* by the following characteristics: head yellow (vs blackish); mid-lobe of mesoscutum without median line (vs vague); median carina of propodeum not forked anteriorly (vs always forked); plicae distinct but not connected with median carina (connected by anterior oblique carinae); forewing SMV with three dorsal setae (vs five).

#### Description.

**Figures 1–4.**
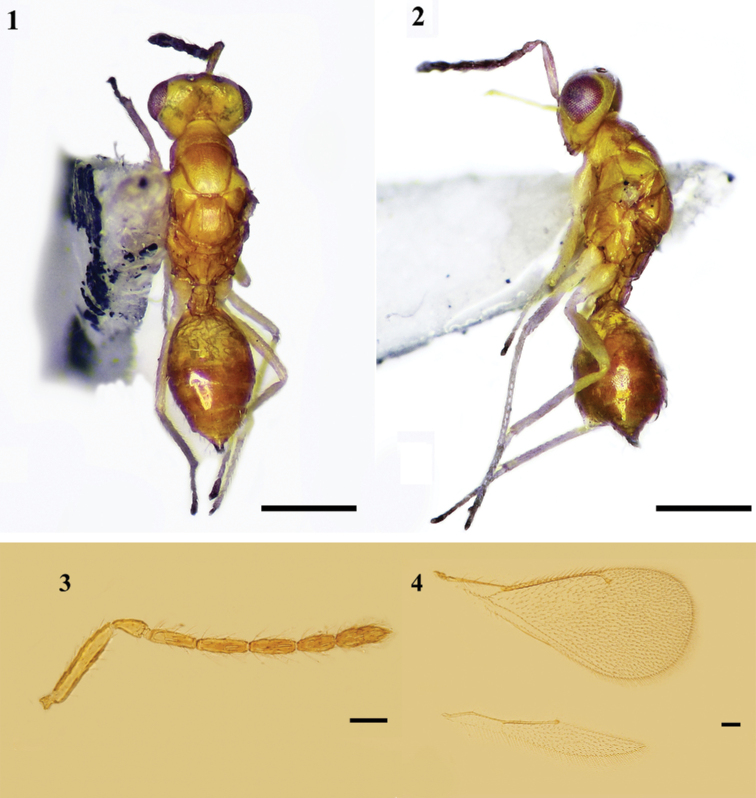
*Mestocharellaqingdaoensis* sp. nov., holotype, female **1** habitus, dorsal view **2** habitus, lateral view. Scale bars: 500 μm **3** antenna, lateral view **4** fore and hind wings, dorsal view. Scale bars: 100 μm.

**Female.** Body length 1.8–1.9 mm, mainly yellow (Figs 1, 2). Head yellow, eyes deep reddish brown, ocellus yellowish white; antenna scape yellowish, pedicel and flagellum yellowish brown. Metasoma mainly brownish with posterior half of mesoscutum and axillae yellow; wings hyaline, venation yellowish brown; legs yellow, tarsomere IV of all legs dark brown. Mesosoma brownish with basal 1/3 yellowish brown.

Head in dorsal view, nearly as broad as mesosoma, 2.5–2.6× (2.5×) as broad as long; vertex with setae shorter than OD, POL 1.3× OOL, OOL 2. × OD. Face depressed slightly, without median line; torulus with lower edge above the ventral edge of eyes; eyes separated by 1.45× their height. Malar sulcus present; malar space 0.6× as long as eye height. Mouth cavity 1.4× as wide as malar space; clypeus with anterior margin bidentate; mandible tridentate. Antenna (Fig. 3) scape 5× as long as broad with shorter setae on dorsal and ventral side; two anelli, first anellus slightly transverse, second anellus lamellar; pedicel 2.2–2.3× as long as broad, shorter than F1; F1–F4: 3.0×, 3.4×, 3.2×, 2.3× as long as broad respectively; clava 3.2× as long as broad, ca as broad as F3, bi-segmented; flagellum with long whorled setae.

Metasoma relatively long, 1.7–1.8× (1.8×) as long as broad. Pronotum subconical, 3.15× as broad as long, ~0.6× as long as mid-lobe of mesoscutum; collar rounded anteriorly and without transverse carina. Mid-lobe of mesoscutum with extremely fine reticulation; without median line; 3 adnotaular setae in one row on each side. Scutellum ca. as long as broad; submedian grooves shallow but distinct enclosing a space ~2.9× as long as broad, sublateral grooves distinct without weak costulae; anterior setae situated before middle distinctly. Dorsellum ~3× as broad as long, with a weak median carina. Propodeum subpentagonal area broad, smooth, without reticulation, median carina distinct and thin, not forked anteriorly; plicae distinct but not connecting with median carina; spiracle small, circular; callus with 2 setae. Forewing (Fig. 4) 2.2× as long as broad, SMV with 3 dorsal setae; costal cell shorter than MV, MV 6.9–7.3× (7.3×) as long as STV with front edge 12–15 setae; STV short with a long uncus; speculum small, nearly closed posteriorly, subcubital line of setae not reaching to distal edge of speculum. Legs slender, spur of metatibia 0.5× as long as length of metabasitarsus.

Gastral petiole long with several transverse weak carinae anteriorly and 3 or 4 longitudinal strong carinae. Gaster 1.2–1.4× as long as broad, shorter than mesosoma; ovipositor 0.5× as long as gaster and slightly exserted at apex of gaster, tip of hypopygium situated at basal 4/5 of gaster.

**Male**. Unknown.

#### Host.

Unknown.

#### Distribution.

China (Shandong).

#### Etymology.

The epithetic *qingdao* refers to the place where the species collected.

### ﻿ Nesolynx

Taxon classificationAnimaliaHymenopteraEulophidae

Ashmead, 1905

683560BF-05BB-5EAF-9398-16D42051B28F

#### Note.

The genus *Nesolynx* was erected by Ashmead (1905) with *Nesolynxflavipes* Ashmead, 1905 as the type species. Bouček (1988) proposed *Aceratoneurella* Girault, 1917, *Ceratotrastichus* Girault & Dodd, 1913, and *Omphalomomyia* Girault, 1913 as synonyms of *Nesolynx*. It is a characteristic genus with 17 species recorded worldwide (Noyes 2019), but only one species, *Nesolynxthymus* (Girault, 1916), is found in China. It is distributed in tropical and subtropical countries, in the warmer parts of the temperate zones of Europe, Africa, Asia, Australia, and the Pacific islands (Bouček 1988). It can be distinguished from Tetrastichinae particularly by the mid-lobe of mesoscutum bearing dense setae and without a median line (Bouček 1988). The species are parasitoids of various groups of Diptera and Lepidoptera (Bouček 1988).

### ﻿ Nesolynx thymus

Taxon classificationAnimaliaHymenopteraEulophidae

(Girault, 1916), new record from China

5CA999FF-1FF9-5363-9821-981CAE6FE2FC

Figs 5–7 , 8–10

Omphalomomyia thymus Girault, 1916: 485.
Omphalomomyia thymus javae Girault, 1917: 7 (subspecies). [Synonymized by Bouček 1977: 404].
Buonapartea aeniceps Girault, 1924: 5. Syntypes. [Synonymized by Bouček 1988: 697].
Syntomosphyrum obscuriceps Ferrière, 1940: 138. [Synonymized by Bouček 1977: 404].
Omphalomonyia [sic] thymus: Thompson 1955: 292.
Nesolynx thymus : Bouček 1977: 404.

#### Material examined.

7 females: [1 female on slide], Henan Province, Xinyang City, Mount Yan, Temple Xianyin, 6–7.VIII.2015, Hui Geng, Zhi-Guang Wu, Yan Gao, by yellow pan trapping; [1 female on slide], Hainan Province, Changjiang County, Mount Bawanglin, 15–17.V.2019, Wen-Jian Li, Jun Wu, by yellow pan trapping; [1 female on slide], Hainan Province, Haikou City, Hainan University, 27–29.VI.2019, Yu-Ting Jiang, by yellow pan trapping; [2 females on cards], Yunnan Province, Yuanjiang County, 26–28.XI.2020, Jun Wu, Jun-Jie Fan; Ming-Rui Li, Gang Fu, by yellow pan trapping; [2 females on cards], Yunnan Province, Shuangjiang County, 21.IV.2013, Xiang-Xiang Jin, Guo-Hao Zu, Chao Zhang, by sweeping. (All deposited in NEFU).

#### Diagnosis.

**Figures 5–7.**
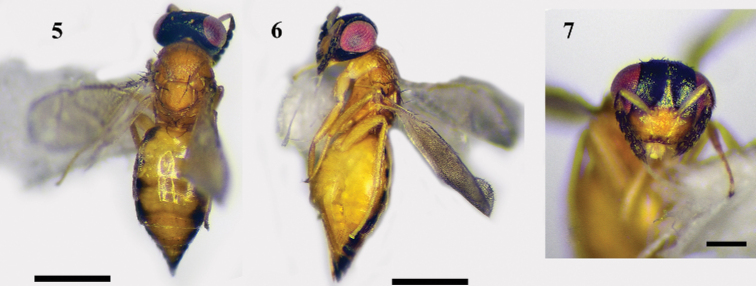
*Nesolynxthymus* (Girault), female **5** habitus, dorsal view **6** habitus, lateral view. Scale bars: 500 μm **7** head, frontal view. Scale bar: 200 μm.

**Female.** Body mainly yellow (Figs 5, 6); upper face, vertex, gena, and occiput dark green with metallic reflections, lower face yellow (Fig. 7); gaster yellow with black sides. Mesosoma with dense setae on mid-lobe of mesoscutum, especially a pair of long black setae posteriorly similar to setae on scutellum; propodeum with median carina distinct, cup-shaped. Gaster 1.6–1.8× as long as broad.

**Male.** Unknown.

#### Hosts.

Not known from China. Non-Chinese records include *Muscadomestica* Linnaeus, 1758, *Exoristabombycis* (Louis, 1880), *Bombyxmori* Linnaeus, 1758 (Bansude et al. 2010), *Argyrophylaxleefmansi* Baranov, 1933, *Bessaremota* (Aldrich, 1925), *Chaetogenabezziana* Baranov, 1934, *Nephantisserinopa* Meyrick, 1905, *Artonacatoxantha* Hampson, 1892 (Herting 1978), *Exoristasorbillans* (Wiedemann, 1830) (Kumar et al. 1991), *Ptychomyiaremota* Aldrich, 1925, *Cadurcialeefmansi* Baranov, 1933 (Lever 1964), *Zaratha* sp. (Bouček 1988), *Sturmiopsisinferens* Townsend, 1916, *Chiloauricilius* Dudgeon, 1905 (Varma 1989), *Cnaphalocrocismedinalis* (Guenée, 1854) (Talgeri and Dalaya 1971), *Marucatestulalis* (Geyer, 1832), Tachinidae unspecified sp. (Narendran 2007), *Apantelesartonae* (Rehwer, 1926) (Herting 1977).

**Figures 8–10.**
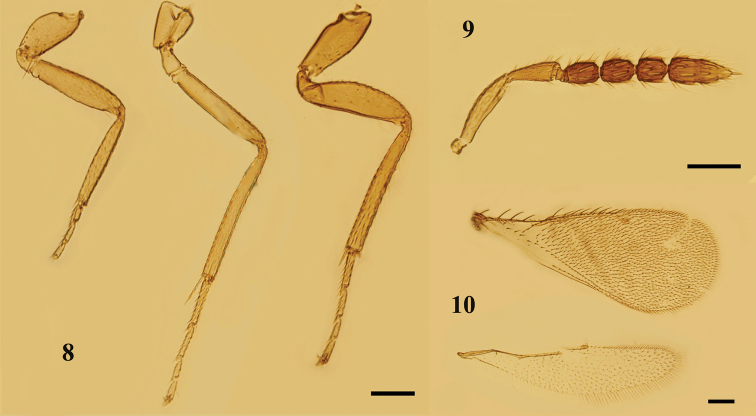
*Nesolynxthymus* (Girault), female **8** legs, lateral view, from left to right: fore, mid, and hind legs **9** antenna, lateral view **10** fore and hind wings, dorsal view. Scale bars: 100 μm.

#### Distribution.

China (Henan, Yunnan, Hainan); Bangladesh (Rahman 1989), Myanmar (Husain and Khan 1986), Indonesia (Bouček 1988), Malaysia (Lever 1964), India, and Sri Lanka (Narendran 2007).

#### Comments.

The species can be easily identified by the unique color of head.

### ﻿ Holcotetrastichus

Taxon classificationAnimaliaHymenopteraEulophidae

Graham, 1987

67ECAE05-5630-5A88-8733-052CA208A99C

#### Note.

This is a small genus erected by Graham (1987), with *Cirrospilusrhosaces* Walker, 1839 as the type species. Only two species have been described: *Holcotetrastichusmanaliensis* Graham, 1991 and *Holcotetrastichusrhosaces* (Walker, 1839). It can be distinguished from other Tetrastichinae especially by the strong transverse costulae in deep broad sublateral grooves and the hypopygium reaching nearly the tip of the gaster (Graham 1987). The species are parasitoids of some species of *Cassida* (Coleoptera, Chrysomelidae) (Graham 1991).

### ﻿ Holcotetrastichus rhosaces

Taxon classificationAnimaliaHymenopteraEulophidae

(Walker, 1839), new record from China

DF67C42D-2F78-52F6-8B75-EC18778FE51F

Figs 11 , 12–17 , 18–20

Cirrospilus rhosaces Walker, 1839: 293.
Cirrospilus racilla Walker, 1839: 312. [Synonymised by Graham 1961: 37].
Tetrastichus racilla : Walker 1848: 149.
Tetrastichus rhosaces : Walker 1848: 147.
Aprostocetus rhosaces : Graham 1961: 37.
Holcotetrastichus rhosaces : Graham 1991: 272; Narendran 2007: 120.
Holcotetrastichus rhosaceus [sic]: Boyadzhiev 2000: 27.

#### Material examined.

9 females and 2 males: [2 females on slides], Liaoning Province, Anshan City, Mount Qianshan, 23.VI.2013, Hui Geng, Zhi-Guang Wu, Yan Gao, Si-Zhu Liu, by sweeping; [1 female on slide], Jiangxi Province, Yichun City, Mount Guanshan, 22–24.VIII.2018, Xiang-Xiang Jin, Wang-Ming Li, by yellow- pan trapping; [1 female and 1 male on slides, 1 female and 1 male on cards], Qinghai Province, Prefecture Huangnan, Forestry Station Maixiu, 26–29.VIII.2019, Ming-Rui Li, by yellow pan trapping; [2 females on cards], Jinlin Province, County Wangqing, Forestry Station Qinhe, 8.VII.2013, Ye Chen, Zhi-Guang Wu, by sweeping; [2 females on cards], Heilongjiang Province, City Heihe, Park Beishang, 22.VII.2020, Ming-Rui Li, by sweeping. (All deposited in NEFU).

**Figure 11.**
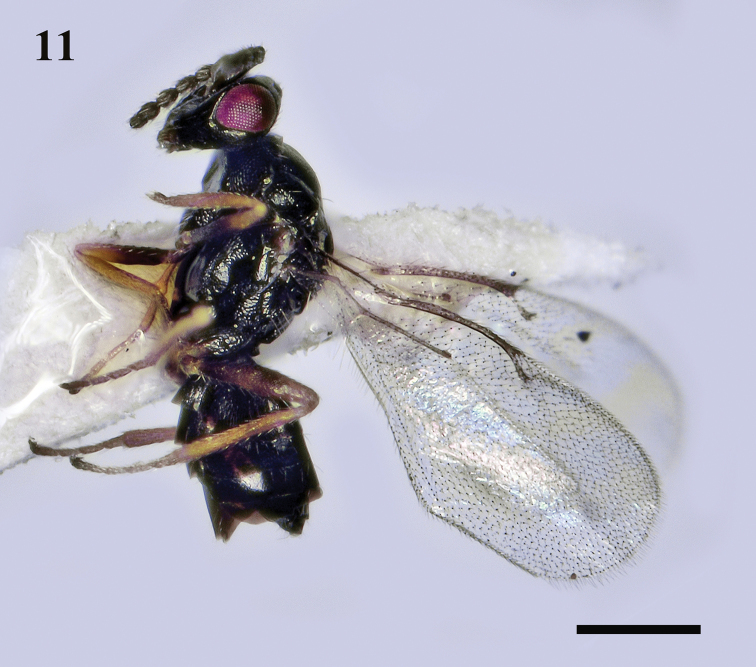
*Holcotetrastichusrhosaces* (Walker), female, habitus, lateral view. Scale bar: 500 μm.

**Figures 12–17.**
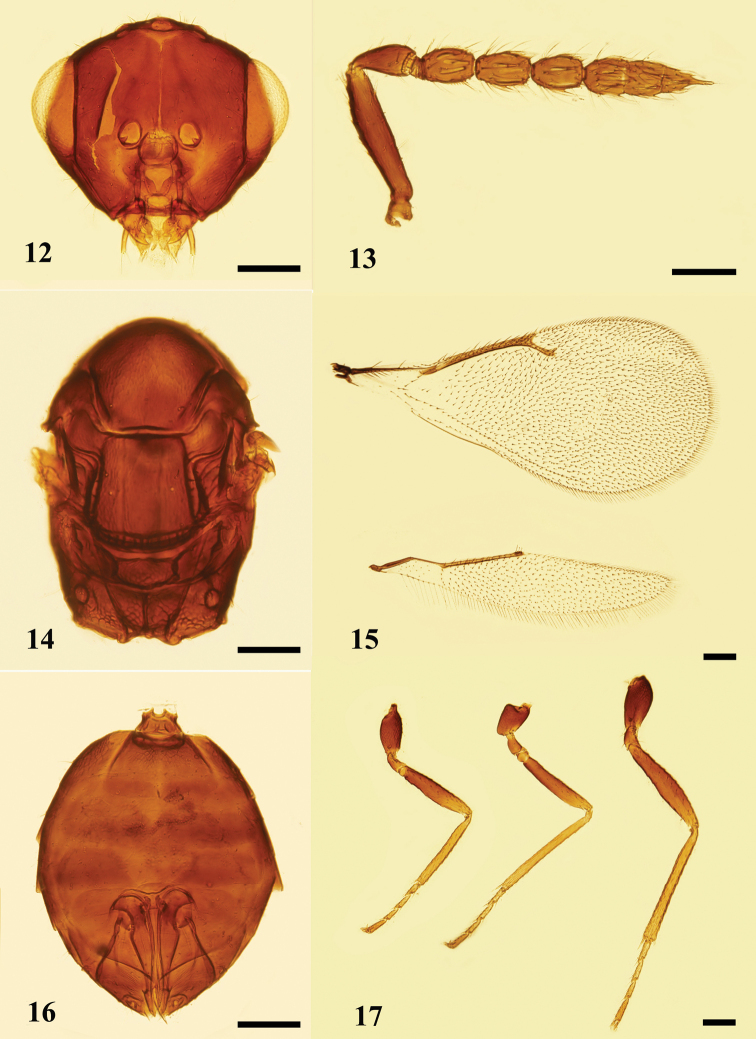
*Holcotetrastichusrhosaces* (Walker), female **12** head, frontal view **13** antenna, lateral view **14** mesosoma, dorsal view **15** fore and hind wings, dorsal view **16** metasoma, ventral view **17** legs, lateral view, from left to right: fore, mid, and hind legs. Scale bars: 100 μm.

**Figures 18–20.**
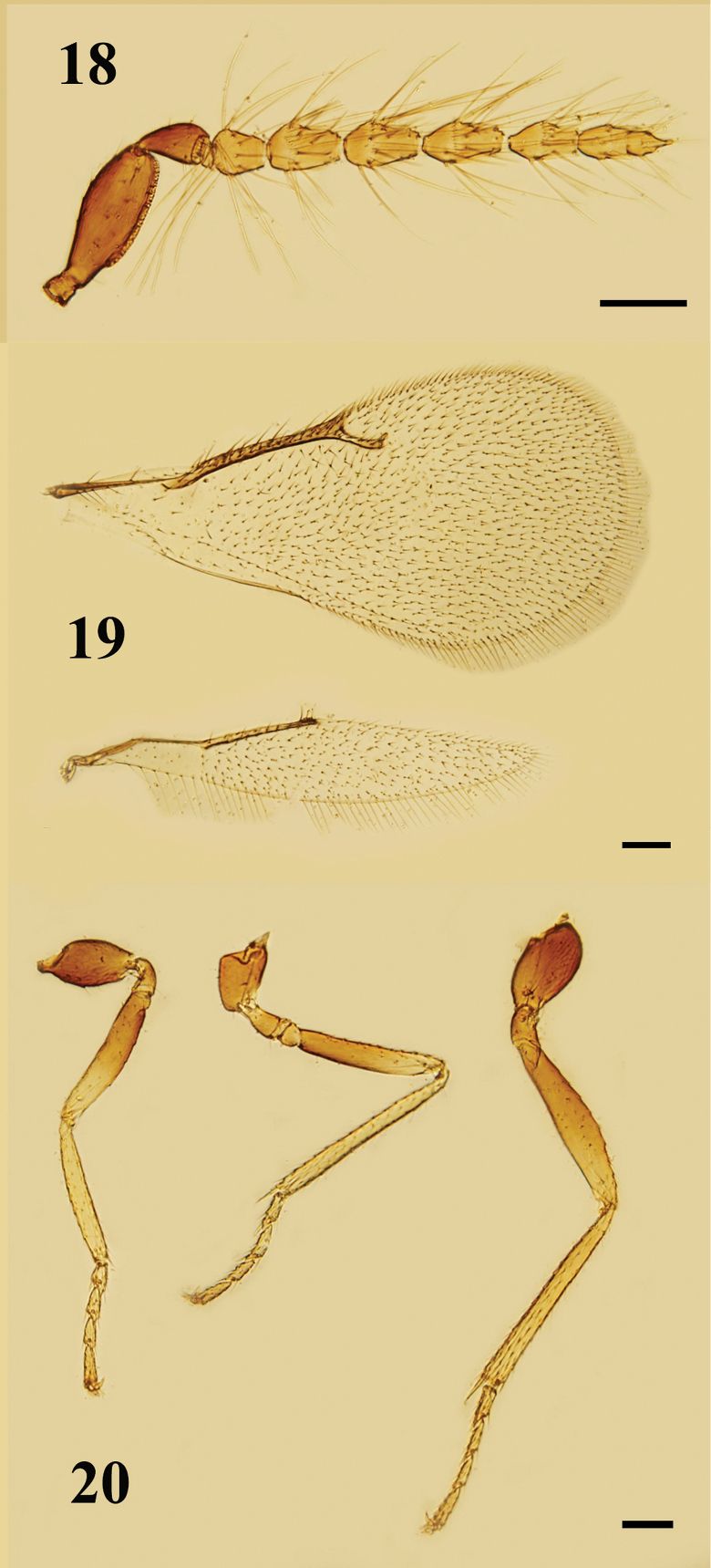
*Holcotetrastichusrhosaces* (Walker), male **18** antenna, lateral view **19** fore and hind wings, dorsal view **20** legs, lateral view, from left to right: fore, mid, and hind legs. Scale bars: 100 μm.

#### Diagnosis.

**Female.** Body black, with weak metallic reflections (Fig. 11). Mesosoma (Fig. 14) with mid-lobe of mesoscutum weakly reticulate, 2 adnotaular setae in single row on each side, median line indicated only posteriorly; sublateral grooves of scutellum deep and broad with strong transverse costulae, submedian grooves rather weak. Forewing (Fig. 15) broad, 2.0× as long as broad, SMV with 2 dorsal setae, MV 2.8–3.2 times length of STV, PMV distinctly short. Gaster (Fig. 16) with hypopygium almost reaching tip of gaster.

**Male.** Antenna (Fig. 18) with scape broad, ventral plaque 0.7 length of scape; F1 shorter than F2; each segment of funicle with whorl setae reaching well beyond the tip of the segment.

#### Hosts.

Unknown from China. Non-Chinese records include *Cassidadeflorate* Suffrian, 1844, *Cassidamurraea* Linnaeus, 1767, *Cassidanebulosa* Linnaeus, 1758, *Cassidanobilis* Linnaeus, 1758, *Cassidarubiginosa* Mueller, 1776, *Cassidaviridis* Linnaeus, 1758, *Cassidavittate* Villers, 1789 (Graham 1991), *Cassidapiperata* Hope, 1842 (Nagasawa et al. 2003).

#### Distribution.

China (Heilongjiang, Liaoning, Jilin, Qinghai, Jiangxi); Austria, Czech Republic, Czechoslovakia, France, Germany, Hungary, Ireland, Italy, Moldova, Romania, Switzerland, United Kingdom (Graham 1991), Bulgaria (Boyadzhiev 2000), Netherlands (Gijswijt 2003), Poland (Domenichini 1966), Russia (Yegorenkova et al. 2007), Sweden (Hansson 1991), Japan (Ikeda 1997), and United States of America (Boyadzhiev 2000).

#### Comments.

Most species we collected had weak metallic reflections compared to the species reported by Graham (1991).

### ﻿ Peckelachertus

Taxon classificationAnimaliaHymenopteraEulophidae

Yoshimoto, 1970

F5065945-69B3-5930-9F71-26AE03CF8F25

#### Note.

This is a small genus with only two known species worldwide (Noyes 2019): *P.diprioni* Yoshimoto, 1970 and *P.anglicus* Graham, 1977. Both of these were transferred from the subfamily Elachertinae to Tetrastichinae by Graham (1977). The genus can be distinguished from other Tetrastichinae especially by having the PMV equally or nearly as long as STV and scutellum without submedian grooves (Graham 1977).

### ﻿ Peckelachertus diprioni

Taxon classificationAnimaliaHymenopteraEulophidae

Yoshimoto, 1970, new record from China

AEBFA5AA-2A4F-5EA8-AA4E-51BD35859B00

Figs 21–26

Peckelachertus diprioni Yoshimoto, 1970: 909.
Peckelachertus diprioni : Graham 1977: 47.

#### Material examined.

2 females. [2 females on slides], China, Heilongjiang Province, Shangzhi City, Mount Laoyeling, 9.VII.2015, Ye Chen, Chao Zhang, by sweeping.

#### Diagnosis.

**Female.** Body dark brown, without metallic reflections. Head with anterior margin of clypeus truncate, without any teeth, malar sulcus present and distinct. Antenna with pedicel 1.8–1.9× as long as broad, F11.6× as long as broad. Mesosoma (Fig. 14) 1.5× as long as broad, mid-lobe of mesoscutum with 2 adnotaular setae in single row on each side, median line absent; scutellum submedian grooves absent or indicated at posterior half, anterior pair of setae situated near anterior margin of scutellum. Forewing (Fig. 15), 2.2× as long as broad, SMV with 4 dorsal setae, the length of PMV as long as STV.

**Figures 21–26.**
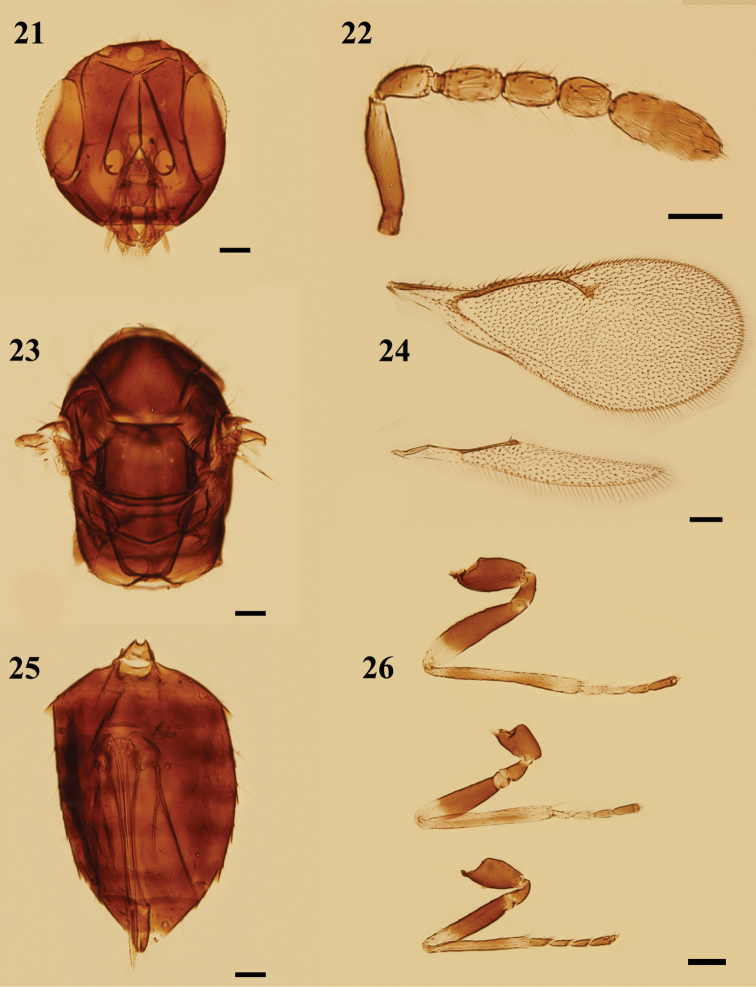
*Peckelachertusdiprioni* Yoshimoto, female **21** head, frontal view **22** antenna, lateral view **23** mesosoma, dorsal view **24** fore and hind wings, dorsal view **25** metasoma, ventral view **26** legs, lateral view, from bottom to top: fore, mid, and hind legs. Scale bars: 100 μm.

**Male.** Unknown from Chinese material.

#### Hosts.

Unknown from China. Non-Chinese records include *Gilpiniafrutetorum* (Fabricius, 1793) (LaSalle 1994), *Gilpiniapallida* (Klug, 1812) (Graham 1977).

#### Distribution.

China (Heilongjiang); Finland, Canada (Graham 1977).

#### Comments.

Graham (1977) pointed out that Yoshimoto’s description of genus *Peckelachertus* and of its type species *P.diprioni* are not correct in some respects and proposed some remarks after examining material. Our specimens agree well with the remarks by Graham (1977).

### ﻿ Ceratoneura

Taxon classificationAnimaliaHymenopteraEulophidae

Ashmead, 1849

969B85CB-A33C-5EDC-9F5D-0B85C140B664

#### Note.

The genus *Ceratoneura* was erected with *Ceratoneurapetiolata* Ashmead, 1894 as the type species by subsequent designation of Ashmead (1904). Ikeda (2001) revised of the world species of *Ceratoneura* in detail, describing five new species and redescribing six known species. It is a small genus with 12 species recorded worldwide (Noyes 2019), but only one species *Ceratoneuraindi* Girault, 1917 has been reported from China (Ikeda 2001). This genus can be distinguished from other Tetrastichinae especially by the strongly sclerotized body and the face with conspicuous striae radiating from the mouth. The species are parasitoids of various groups of Diptera and Lepidoptera (Bouček 1988).

### ﻿ Ceratoneura indi

Taxon classificationAnimaliaHymenopteraEulophidae

Girault, 1917

5618D15E-9A74-540E-A999-DE76C3A7BC4D

Figs 27–28 , 29–31

Ceratoneura indi Girault, 1917: 10.
Ceratoneura indica Rohwer, 1921: 127. [Synonymized by Bouček 1988: 670].

#### Material examined.

7 females: [1 female on slide and 2 females on cards], China, Zhejiang Province, County Panan, Mount Dapan, 30.VI. –2.VII.2019, Jun Wu, Jun-Jie Fan, by yellow pan trapping; [4 femles on cards], China, City Chongqin, Mount Simianshan, Village Hongdong, 26.VII.2019, Ting-Ting Zhao, Shu-Cheng Deng, by sweeping. (All deposited in NEFU).

**Figures 27, 28.**
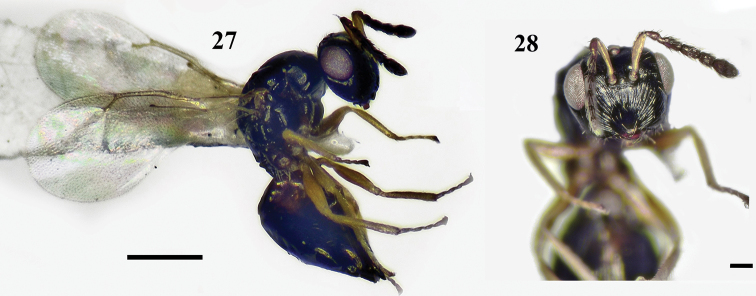
*Ceratoneuraindi* Girault, female **27** habitus, lateral view. Scale bar: 500 μm **28** head, frontal view. Scale bar: 100 μm.

**Figures 29–34.**
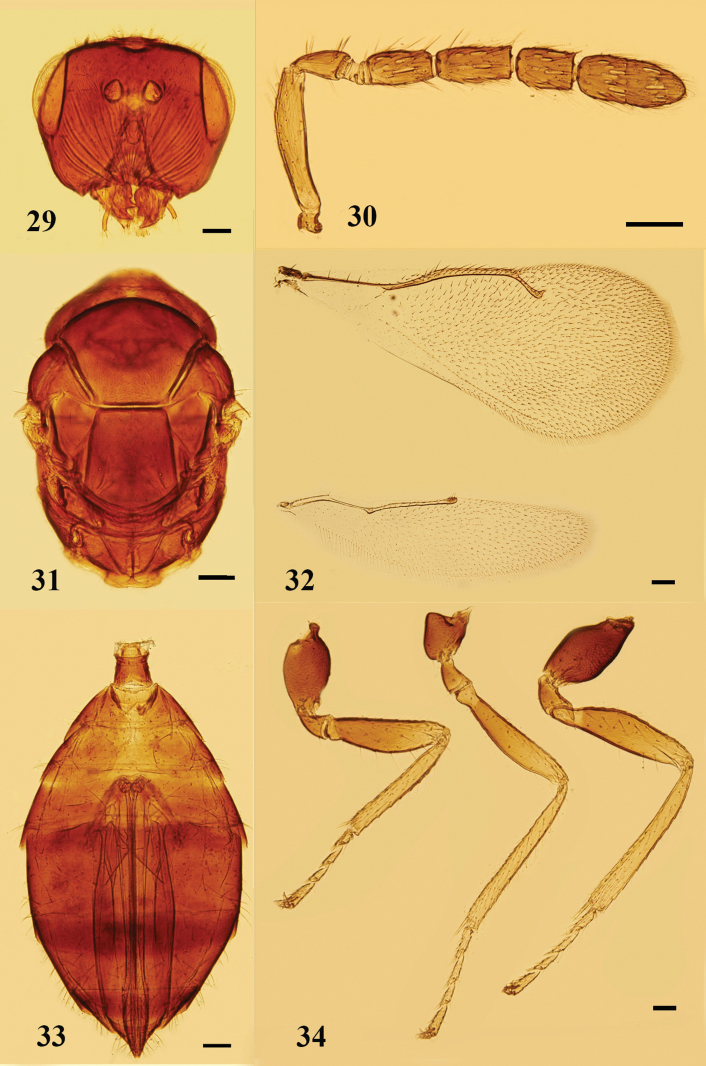
*Ceratoneuraindi* Girault, female **29** head, frontal view **30** antenna, lateral view **31** mesosoma, dorsal view **32** fore and hind wings, dorsal view **33** metasoma, ventral view **34** legs, lateral view, from left to right: fore, mid, and hind legs. Scale bars: 100 μm.

#### Diagnosis.

**Female.** Body black, strongly sclerotized. Face with conspicuous striae radiating from mouth, torulus with lower margin distinctly above the level of ventral margin of eyes. Mesosoma with mid-lobe of mesoscutum weakly reticulate, 4 or 5 adnotaular setae in single row on each side, median line absent. Forewing 2.2–2.3× as long as broad, SMV with 3 dorsal setae, speculum large. Petiole distinct, 0.4–0.5× as long as propodeum. Gaster 1.7–2.0× as long as broad.

**Male.** Unknown for Chinese material.

#### Hosts.

Unknown from China. Non-Chinese records include *Asphondyliasphaera* Monzen, 1937 (Ikeda 2001).

#### Distribution.

China (Zhejiang, Chongqing, Hong Kong), Japan, India, Malaysia, New Caledonia, Papua New Guinea, Sri Lanka.

#### Comments.

Ikeda (2001) reported only one specimen from Hong Kong, and we add seven additional specimens from Zhejiang and Chongqing, which are new locality records for China.

## Supplementary Material

XML Treatment for Mestocharella qingdaoensis

XML Treatment for Nesolynx

XML Treatment for Nesolynx thymus

XML Treatment for Holcotetrastichus

XML Treatment for Holcotetrastichus rhosaces

XML Treatment for Peckelachertus

XML Treatment for Peckelachertus diprioni

XML Treatment for Ceratoneura

XML Treatment for Ceratoneura indi
